# Purulent pericarditis and cardiac tamponade in HIV: a case report on a dreaded complication of *Streptococcus pneumoniae*

**DOI:** 10.1097/MS9.0000000000002552

**Published:** 2024-09-10

**Authors:** Laxman Wagle, Parmartha Basnyat, Anuj Timshina, Rashmita Regmi, Lakpa Diku Sherpa, Sishir Poudel

**Affiliations:** a Department of Internal Medicine, Ascension Saint Agnes Hospital, Baltimore, MD, USA; b Department of Internal Medicine, Patan Academy of Health Sciences, Kathmandu; c Department of Nursing, Karnali Academy of Health Science, Jumla; d Department of Internal Medicine, B.P. Koirala Institute of Health Science, Dharan, Nepal

**Keywords:** cardiac tamponade, HIV, pericarditis, purulent pericarditis, Streptococcus pneumoniae

## Abstract

**Introduction and importance:**

Purulent pericarditis is an uncommon complication of *Streptococcus pneumoniae*, which commonly occurs in an immunocompromised state such as HIV and can lead to life-threatening complications such as cardiac tamponade and potentially death if untreated. Early identification, pericardiocentesis, and general measures such as antibiotics and anti-inflammatory medications can be life-saving.

**Case presentation:**

The authors present a case of a 64-year-old male with HIV who presented with clinical symptoms suggestive of pericarditis. Chest imaging revealed multifocal airspace diseases and moderate pericardial effusion. He had worsening lactic acidosis, and bedside point-of-care ultrasound showed pericardial effusion with features suggestive of cardiac tamponade. His lactic acidosis improved with emergency pericardiocentesis. Blood and pericardial fluid cultures revealed *Streptococcus pneumoniae*. He was further treated with intravenous antibiotics, colchicine, and ibuprofen.

**Clinical discussion:**

Although *Streptococcus pneumoniae* is a common etiology of community-acquired pneumonia (CAP), it has not been cited as the leading cause of pericarditis or pericardial effusion. In immunocompromised patients, it is necessary to consider a broad differential diagnosis as an etiology of acute chest pain, as it may be challenging to differentiate pleuritic and pericarditic chest pain from clinical presentation only. Moreover, infectious etiology of acute pericarditis and pericardial effusion should be considered in this patient population, especially those with HIV. At the same time, it is crucial to promptly identify and treat cardiac tamponade to prevent further deterioration.

**Conclusion:**

This case provides insight into the diagnosis and management of CAP and its potential complication of purulent pericarditis and cardiac tamponade in immunocompromised patients.

## Introduction

Highlights

- Purulent pericarditis, a rare but severe complication of *Streptococcus pneumoniae* infection, often occurs in immunocompromised individuals, such as those with HIV. This condition can lead to life-threatening outcomes like cardiac tamponade if not promptly treated. The early identification of symptoms, combined with interventions such as pericardiocentesis, antibiotics, and anti-inflammatory medications, is crucial for survival. *Streptococcus pneumoniae* is a well-known cause of community-acquired pneumonia (CAP), but it is not commonly associated with pericarditis or pericardial effusion. This case report highlights the critical need for vigilant diagnosis and management in immunocompromised patients presenting with CAP to prevent complications like purulent pericarditis and cardiac tamponade.
- The case involves a 64-year-old HIV-positive male with a history of non-compliance with anti-retroviral therapy, who presented with symptoms indicative of acute pericarditis, including severe chest pain and shortness of breath. Diagnostic imaging and laboratory findings confirmed purulent pericarditis and cardiac tamponade, necessitating emergency pericardiocentesis. The cultures identified Streptococcus pneumoniae as the causative agent. The patient’s treatment included broad-spectrum antibiotics, colchicine, and ibuprofen, alongside HIV management. This case underscores the importance of early detection and comprehensive treatment strategies for purulent pericarditis in patients with CAP, particularly those with underlying immunocompromised conditions, to mitigate the risk of severe complications and improve outcomes.

Community-acquired pneumonia (CAP) is a lung infection contracted in the community caused by bacterial, viral, and, less commonly, fungal or parasitic agents. Lower respiratory tract infections, such as CAP, are a frequent health concern worldwide and are responsible for 4.7% of all deaths, according to the WHO^1^. Studies have identified *Streptococcus pneumoniae* as the leading cause (20%) of CAP, which requires hospitalization or outpatient care, followed by *Haemophilus influenzae* (10.8%) and *Mycoplasma pneumoniae* (7.5%)^2^. Since the advent of antibiotics, the prognosis and management of pneumonia have generally been favorable. However, complications such as empyema, pericarditis, and endobronchial obstruction can still arise, possibly culminating in fulminant disease if purulent pericarditis occurs^3^. Gentile *et al.* ^4^ found in their longitudinal study an incidence rate of 2.1 cases of bacteremic pneumococcal pneumonia (BPP) per 1000 admissions for CAP with a mean patient age of 60 years, and comorbidities were found in 65% of the cases. In the United States, among adults younger than 65 years old, the incidence of community-acquired pneumonia (CAP) ranges from 24.8 to 106 cases per 10 000 person-years^5,6^.

Acute pericarditis is characterized by chest pain, shortness of breath, and a pericardial friction rub accompanied by electrocardiographic changes and elevation of cardiac biomarkers, reflecting acute inflammation of the pericardium. Although a specific etiology cannot be established in most instances of acute pericarditis, viral infections are considered the culprit in most cases^7^. Other infectious etiologies, such as tuberculosis, are common in developing countries. Bacterial or fungal pericarditis is less common but can occur in an immunocompromised state, as in human immunodeficiency virus (HIV) patients, and can potentially lead to fulminant disease and cardiac tamponade. A proposed mechanism for S. pneumoniae pericarditis following CAP involves both hematogenous spread from a distal infection and translocation of S. pneumoniae into the pericardium. Incident cardiac complications that occur during CAP independently affect short-term mortality^8^.

## Case presentation

We present the case of a 64-year-old male with HIV, non-adherence to anti-retroviral therapy, and opioid use disorder managed with methadone who presented to the emergency room with complaints of substernal chest pain, fever, cough with yellowish sputum, shortness of breath, palpitations, and fatigue for 1 week. Chest pain was sharp, localized in the substernal region, rated 10/10 in intensity, non-radiating, and exacerbated by coughing, sitting up, and walking, with no alleviating factors noted. He denied experiencing nausea, vomiting, urinary complaints, diarrhea, recent contact with sick individuals, or travel. Upon examination, the vitals were stable, but in mild distress due to chest pain. Tenderness was observed along the chest wall, particularly in the substernal and epigastric regions. White patches and plaques were observed on the tongue. Auscultation revealed crackles in the right lung, but there was no evidence of a pericardial rub in the cardiac region. The remaining examinations did not yield any significant findings.

**Figure 1:**
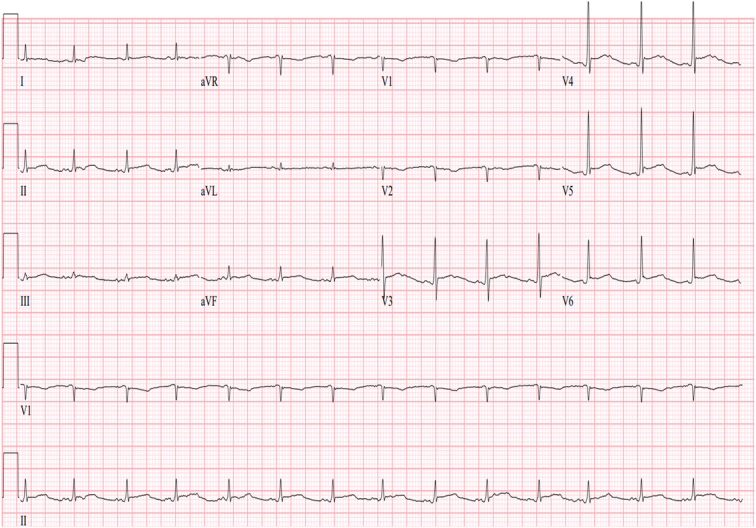
ECG showing ST elevation in inferior and lateral leads.

**Figure 2:**
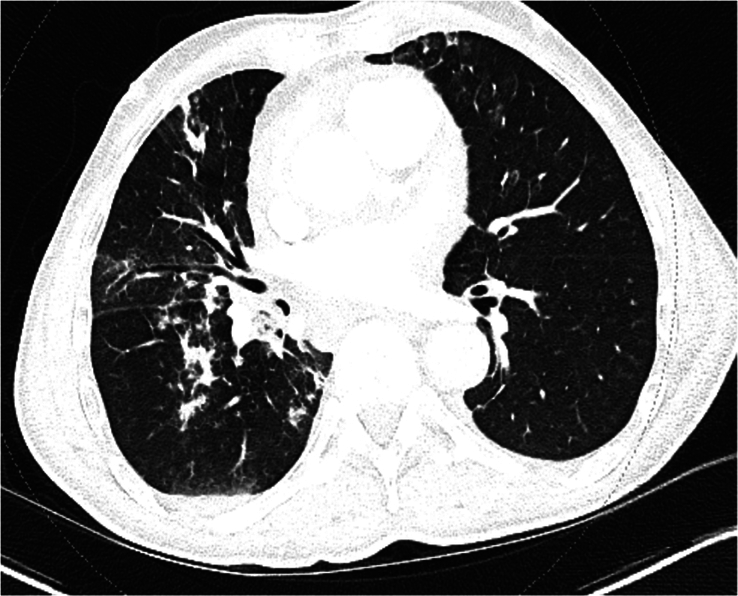
Showing right middle lobe patchy opacities.

**Figure 3:**
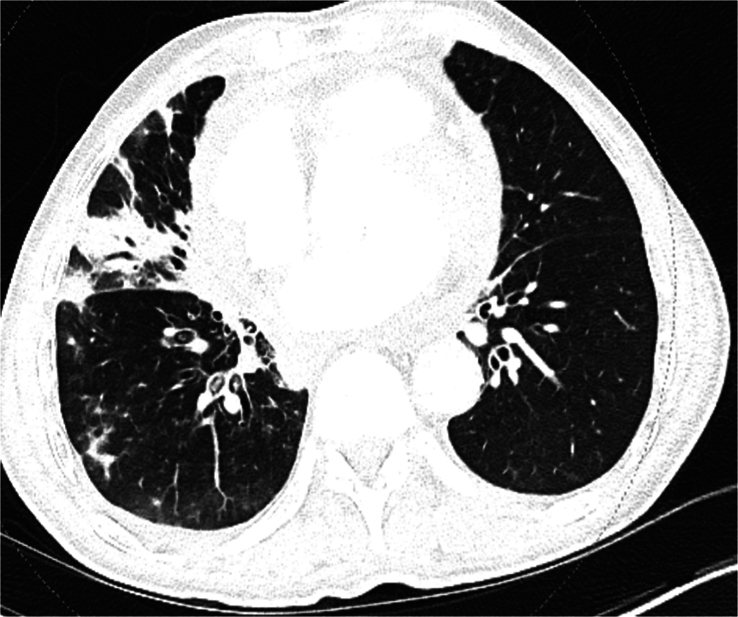
Showing right middle lobe patchy opacities.

Laboratory findings showed neutrophilic leukocytosis of 18 000, a normal basic metabolic profile, and liver function tests. The initial high-sensitivity troponin I level was 110, and the follow-up troponin level was 103 after 1 hour (normal <35). Electrocardiography (EKG) showed normal sinus rhythm with ST-segment elevation in the inferior and lateral leads with PR depression (Fig. 1). Chest radiography revealed infiltrates suggestive of atelectasis or scarring on the right lung bases. Computed tomography (CT) angiography of the chest revealed multifocal airspace diseases and moderate pericardial effusion (Fig. 2 and 3).

The patient was initially admitted for sepsis secondary to pneumonia, given the presence of fever, cough, leukocytosis, and findings in the chest imaging. Venous gas analysis revealed metabolic acidosis with a pH of 7.30, bicarbonate level of 19, and elevated lactic acid levels of 6.3 mmol/l (Normal <1.6 mmol/l). Broad-spectrum antibiotics and intravenous fluids were administered to the patient. A repeat lactic acid measurement after 3 h showed further elevation to 7.4 mmol/l even after the bolus of intravenous fluids (30 ml/kg). The subsequent monitoring revealed a worsening trend of lactic acidosis up to 9 mmol/l. A bedside point-of-care ultrasound was performed, which showed moderate-to-large pericardial effusion with diastolic right atrial and ventricular collapse consistent with cardiac tamponade (Video 1, Supplemental Digital Content 1, http://links.lww.com/MS9/A601). Emergency pericardiocentesis was performed with the placement of a pericardial drain, and 350 ml of cloudy yellow fluid was removed. Following pericardiocentesis, the lactic acid level decreased to 2.4 mmol/l.

Blood and pericardial fluid cultures were positive for *S. pneumoniae*. With *S. pneumoniae* identified as the causative agent in conjunction with clinical findings and diagnostic investigations indicative of purulent pericarditis, he was started on colchicine, ibuprofen, and intravenous antibiotics. Intravenous antibiotics were continued, and the arrangement was made to complete a total duration of four weeks after post-negative cultures due to a low CD4 count (CD4 count 59). He was also started on trimethoprim-sulfamethoxazole prophylaxis, and anti-retroviral therapy was re-initiated. The patient showed clinical improvement following a multi-modal treatment approach. He was scheduled for a follow-up visit with a cardiologist and infectious disease specialist on discharge. His symptoms did not recur on follow-up in 3 months.

## Discussion

Acute pericarditis, as a complication of CAP, has become rare since the advent of antibiotics. However, such cases still occur. In a study done among 933 patients diagnosed with acute pericarditis, the etiologies were idiopathic in 55%, autoimmune or post-cardiac injury syndromes (24%), neoplastic (9%), bacterial (3.1%), and tuberculosis (0.5%)^7^. In the modern era, bacterial pericarditis has decreased with an incidence of 1/18 000^9^. Pericarditis accounts for 5% of emergency room admissions for chest pain without acute myocardial infarction^10^. In individuals with immunocompromised states, such as those with HIV, it is imperative to conduct a thorough assessment when presented with acute chest pain.

Distinguishing between pleuritic and pericarditic chest pain based solely on clinical presentation can be challenging, underscoring the need for a comprehensive evaluation to rule out potentially life-threatening conditions like pericarditis and cardiac tamponade. Our patient was initially started on treatment for pneumonia, given the presence of fever, cough, leukocytes, and infiltrates in the right middle lobe. The chest pain was initially thought to be pleuritic. Other life-threatening conditions that could present with pleuritic chest pain, such as pulmonary embolism, aortic dissection, or tension pneumothorax, were ruled out based on CT angiography of the chest. EKG finding of ST-segment elevation in the inferior and lateral leads with PR depression hinted at a diagnosis of acute pericarditis that could also explain the worsening of the clinical condition of the patient with the development of the pericardial effusion and cardiac tamponade.

Purulent pericarditis is a rare but often fatal cause of acute pericarditis, arising from intrathoracic infections, hematogenous dissemination, or direct infection due to trauma or surgical procedures. However, purulent pericarditis has a poor prognosis and frequently leads to complications, such as constrictive pericarditis^11^. Pericardial effusion is a common cardiac complication in patients with HIV, with incidence up to 11%, and tuberculosis is often the etiology in the endemic areas^12,13^. Literature reviews done in the past have shown that 11-17% of cases of cardiac tamponade in HIV patients were caused by bacterial organisms^14,15^. The most common bacteria besides Mycobacterium responsible for purulent pericarditis include *Staphylococcus aureus* and *Streptococcus pneumoniae*. It has been noted that most of the pericardial effusions occurred in patients with AIDS, and these patients also had significantly low CD4 counts (68±74/mm^3^) and a reduction in survival (36% at 6 months as compared to those without pericardial effusion whose survival was 93% at 6 months)^13,16^. Moreover, purulent pericarditis usually occurs in HIV patients along with concurrent bloodstream infections or other fulminant infections.

Our HIV-positive patient, who was non-adherent to anti-retroviral therapy, presented with severe substernal chest pain typical of acute pericarditis. According to the European Society of Cardiology (ESC) guidelines, a detailed medical history of chest pain, typically sharp and pleuritic, improved by sitting up and leaning forward, physical examination with emphasis on heart auscultation (pericardial friction rub), chest radiography electrocardiogram, echocardiography, and routine blood tests (mainly CRP), troponin, and thyroid hormones are required for the evaluation of patients suspected with acute pericarditis^17^.

The American Society of Echocardiography suggests utilizing elevated C-reactive protein or late gadolinium enhancement on cardiac magnetic resonance imaging (CMR) as additional confirmatory markers for pericarditis and echocardiography, cardiac CT, and CMR continues to be widely employed as complementary imaging techniques for diagnosis. In our patient’s case, CT angiography of the chest revealed multifocal airspace diseases along with moderate pericardial effusion. Echocardiography remains the primary imaging modality for diagnosis, aiding in differentiating acute pericarditis from myocardial ischemia by excluding specific wall motion abnormalities in patients with acute chest pain, although ~5% of cases with myocardial involvement still exhibit such abnormalities^17^. It can be carried out in emergency or bedside settings, enabling the integration of cardiac structure and function with Doppler for hemodynamic assessment, an approximate estimation of the size of pericardial effusion, evaluation for myocarditis as well as assist in pericardial drainage^18,19^. However, it is subject to limitations such as restricted imaging windows and reliance on operator skills. On the other hand, CT scans offer rapid acquisition times, dependable evaluation of pericardial thickening and calcifications, and a wide field of view for detecting non-cardiac issues, as in our case. Nevertheless, they do not provide information on ventricular or valvular function. While offering superior tissue characterization and excellent temporal resolution for identifying rapid hemodynamic processes and late gadolinium imaging for identifying ongoing inflammation and pericardial masses, Cardiac MRI may not always be as readily accessible as the other methods^20^. Therefore, CT scan and CMR are recommended as adjuvant for evaluating pericarditis when findings from echocardiography are ambiguous or there is suspicion of hemopericardium, malignancy, trauma, or extracardiac involvement^18^.

Notably, point-of-care ultrasound in our patient revealed moderate-to-large pericardial effusion with diastolic collapse of the right atrium and ventricle, suggestive of cardiac tamponade. The early identification of such acute and fatal complications can allow for early intervention to prevent mortality. Emergent pericardiocentesis is needed if there is evidence of cardiac tamponade or hemodynamic instability. Moreover, pericardiocentesis can be therapeutic and diagnostic for suspected purulent pericarditis; however, pericardial fluid analysis may not yield a specific organism in many cases. In contrast to our case, where a specific microbial etiology was identified, most pericarditis cases are idiopathic and may not require immediate admission or intensive monitoring. However, certain clinical features, including fever above 38°C, a subacute presentation, significant effusion or tamponade, and resistance to aspirin or NSAID therapy, indicate a heightened risk of underlying causes and complications^21^. In such cases, admission and further evaluation are warranted to ensure appropriate management. In cases of sepsis, tuberculosis, or HIV-positive infection, bacterial cultures can provide diagnostic clarity, as recommended by the ESC guidelines. The blood culture of our patient with suspected sepsis showed the presence of Streptococcus pneumoniae. This bacterium was also found in the pericardial fluid culture. Notably, the in-hospital mortality rate for acute pericarditis is relatively high (1.1% as per a study) and increases significantly with an increase in age and the presence of concurrent severe infections^22^. Prompt diagnosis and treatment of purulent pericarditis are crucial, particularly in patients with multiple underlying comorbidities and infections.

The 2015 ESC guidelines recommend using colchicine with NSAIDs/aspirin for three months for acute pericarditis and six months for recurrent pericarditis. CRP levels should guide treatment duration and response^17^. American College of Cardiology (ACC) recommends tapering NSAIDs in 2–4 weeks after resolution of symptoms of acute pericarditis and giving colchicine for three months in acute pericarditis and for 6 months in recurrent pericarditis at a dose of 0.5–1.2 mg per day^19^. However, it should be noted that further investigation and treatment for a specific etiology are warranted if clinical symptomatology does not improve with anti-inflammatory medication. A course of antimicrobial therapy targeted against the causative bacteria is recommended in cases of purulent pericarditis. Intrapericardial fibrinolytics have also shown efficacy in fibrinopurulent pericarditis, halting drainage without complications and averting pericardial constriction^23,24^. Restriction of intense exercise has been advised by ACC for at least a duration of 3 months after an episode of acute pericarditis^19^.

In the presence of viremia, it is recommended to start anti-retroviral therapy (ART) in HIV-positive patients. Patients on ART have a lower incidence of purulent pericarditis, including tuberculous pericarditis and dilated cardiomyopathy, but face higher cardiovascular risks from obesity, hypertension, and anemia. However, caution should be taken to avoid potential drug-drug interaction between ARTs and antimicrobials (rifamycin and others), and it may be wise to delay initiation of ART in such cases to better understand the side effect profile of the medications, if any but no such clear guideline is in place. Corticosteroids are not recommended as first-line therapy for management of purulent pericarditis. In a multicenter study done in Africa among patients with tuberculous purulent pericarditis, prednisolone reduced the risk of composite outcomes, cardiac tamponade, and constriction but increased the mortality risk. Also, ART lowered the risks of composite outcomes and death but slightly increased the risk of cardiac tamponade and constriction, as per this study. Combining prednisolone with ART may improve outcomes and reduce severe cardiac events in these patients^25^. However, the use of steroids in patients with acute pericarditis has been shown to prolong the disease as well as increase the recurrence. Hence, corticosteroids should not be used in these patients unless they are associated with certain conditions like autoimmune disease or pregnancy^19^.

Recurrent pericarditis affects up to 30% of patients without colchicine treatment, and constrictive pericarditis is rare in idiopathic cases (<1%). Still, it can occur in up to 30% with bacterial or tuberculosis causes, and cardiac tamponade is uncommon in idiopathic cases but more frequent with malignancies and infections^11,26,27^. In a study by Imazio *et al.* ^11^, the incidence of constrictive pericarditis per 1000 person-years was 0.76 for idiopathic/viral etiologies, 4.40 for connective tissue disease/pericardial injury, 6.33 for neoplastic, 31.65 for tuberculous, and 52.74 for purulent pericarditis. Our patient continued anti-retroviral medication, with trimethoprim-sulfamethoxazole added for *Pneumocystis jirovecii* prevention, owing to a low CD4 count. Symptoms gradually improved with antibiotics, colchicine, and ibuprofen therapy. While there are no specific recommendations on follow-up and monitoring, performing a follow-up echocardiogram within the first 3 months or earlier if any symptoms recur is wise. A serial echocardiography study done by Blanchard *et al.* ^16^ showed that left ventricular dysfunction in AIDS patients with pericardial effusion was associated with poor prognosis and 100% mortality in 1 year. Hence, initiation and compliance with anti-retroviral therapy are pivotal in preventing opportunistic or fulminant infections like purulent pericarditis in patients with HIV^28^. Once it occurs, we should have a high suspicion of fatal complications. Adequate treatment (dose and duration) of purulent pericarditis with antimicrobials, NSAIDs, and colchicine, as well as duly performance of pericardiocentesis, can help alleviate the symptoms or prevent a recurrence. Medications such as corticosteroids or anakinra may prove helpful in persistent or recurrent pericarditis and prevent complications such as constrictive pericarditis^29,30^. Further studies are needed to justify the use of these medications in pericarditis.

## Conclusion

This case underscores the importance of considering rare but serious complications, such as purulent pericarditis, in patients with community-acquired pneumonia, especially those with predisposing factors such as HIV infection and medication non-compliance. Recognizing clinical red flags early, such as persistent chest pain and worsening metabolic acidosis, allows for timely diagnostic interventions such as pericardiocentesis. The management approach, guided by ESC guidelines, involved a multidisciplinary effort encompassing broad-spectrum antibiotics, colchicine, ibuprofen, and supportive measures. This highlights the evolving landscape of infectious complications and stresses the need for vigilance and comprehensive management strategies to reduce the morbidity and mortality associated with purulent pericarditis in the context of community-acquired pneumonia.

## Ethical approval

Ethical approval was not required for this study in accordance with local or national guidelines.

## Consent

Written informed consent was obtained from the patient for publication of this case report and accompanying images. A copy of the written consent is available for review by the Editor-in-Chief of this journal on request.

## Source of funding

Not applicable

## Author contribution

L.W.: conceptualization, supervision, investigations, validation, visualization, writing—original draft, writing—review and editing. P.B.: conceptualization, supervision, validation, writing—original draft, writing—review and editing. A.T.: conceptualization, validation, visualization, writing—original draft, writing—review and editing. R.R.: supervision, validation, visualization, writing—original draft, writing—review and editing. L.D.S.: conceptualization, supervision, writing—original draft, writing—review and editing. S.P.: conceptualization, supervision, validation, writing—review and editing.

## Conflicts of interest disclosure

The authors declare no conflicts of interest.

## Research registration unique identifying number (UIN)

Not applicable.

## Guarantor

Laxman Wagle.

## Data availability statement

Not applicable.

## Provenance and peer review

Not commissioned, externally peer-reviewed.

## Supplementary Material
